# Correlation Between Circulating Cell-Free DNA Levels and Breast Cancer Subtypes: A Prospective Observational Study

**DOI:** 10.7759/cureus.42247

**Published:** 2023-07-21

**Authors:** Pushpanjali P, J. R Keshari, Pritam Prakash, Manish Kumar, Manish Mandal, Rekha Kumari

**Affiliations:** 1 Biochemistry, Indira Gandhi Institute of Medical Sciences, Patna, IND; 2 Surgical Oncology, Indira Gandhi Institute of Medical Sciences, Patna, IND; 3 Surgical Gastroenterology, Indira Gandhi Institute of Medical Sciences, Patna, IND

**Keywords:** triple-negative breast cancer, human epidermal growth factor, estrogen receptor-positive, prognostic marker, circulating tumour dna, breast cancer

## Abstract

Introduction: Breast cancer (BC), a heterogeneous disease, is one of the leading causes of cancer-related deaths among women worldwide. Circulating cell-free DNA (cfDNA) levels have been persistently reported to be elevated in BC patients. In the current study, we evaluated the correlation between the cfDNA levels in patients with BC and its subtypes.

Methods: We recruited newly diagnosed, histopathologically confirmed BC patients aged >18 years (N=39), who did not have any previous malignancy, from the Department of Surgical Oncology, Indira Gandhi Institute of Medical Sciences (IGIMS), Patna, Bihar, India. A total of 6 ml of venous blood was collected from each subject; of this, 1 ml was subjected to complete blood count (CBC), and 4 ml was transferred to a clot-activated collection vial for plasma separation and the cfDNA isolation thereof. In addition to the basic demographic history of each patient, the information on the cancer subtype was as also recorded from the medical records of each patient. All the data were analysed by GraphPad Prism Version 8 (Insightful Science, LLC, San Diego, California, United States). One-way ANOVA was used to test the difference between more than two groups. Pearson correlation was also estimated between cfDNA levels and various CBC indices. A two-tailed p-value<0.05 was considered statistically significant.

Results: The mean age of included patients was 48.6±8.20 years. The mean levels of cfDNA were 2.81±2.39 ng/µL. The mean counts of various blood cell types and other indices of CBC were in the normal range. Compared to BC patients with estrogen receptors (ER+), the cfDNA levels were significantly higher in patients with human epidermal growth factor receptor 2 (HER2+) and triple-negative BC (TNBC) (p<0.05).

Conclusion: The elevated levels of cfDNA in patients with BC can be a prognostic marker for the disease subtype. However, more replicative studies are warranted to substantiate our findings.

## Introduction

Breast cancer (BC), a heterogeneous disease, is one of the leading causes of cancer-related deaths among women worldwide. Globally, there are more than 2.3 million new BC cases, resulting in an estimated 650,000 deaths each year [1]. Despite substantial advancements in the therapeutic interventions for BC, the absence of reliable methods for its early detection has been a significant clinical challenge so far. Unlike the subjects with nodal involvement, in BC patients with a localized disease without any nodal involvement, the five-year survival rate is 99%. However, despite advancements in diagnostics, the early detection of the disease has not changed much over the last two decades. Moreover, with high-cost detection methods and varied presentation of the disease, the prevalence of BC-related deaths is higher in developing countries, especially in rural areas [2].

Similar to many cancers, the detection of BC at an early stage is potentially curable [3]. However, with BC being asymptomatic, its early detection and diagnosis remain pivotal for reducing cancer-related deaths [4]. While mammography is a standard care in screening BC, its false-positive and false-negative results have necessitated looking for some other sensitive tools for the early detection of the disorder. Moreover, unnecessary radiation exposure to false positives and delayed treatment for false negative subjects has also been a serious concern in the management of BC. Owing to this, clinicians have been propounding to revisit the standard methods for early detection of BC utilizing modern testing approaches to improve the accuracy of the detection and management of the disease [5].

Among a few, one of the potential biomarkers for BC is the circulating tumour DNA (ctDNA) [6]. The ctDNA refers to small fragments of DNA released into the blood by the necrotic and apoptotic tumour cells. CtDNA is present as a major constituent in the circulating cell-free DNA (cfDNA). The cfDNA estimation and analysis in the peripheral blood offer a minimally invasive method for monitoring disease progression and the effectiveness of therapy [7]. Based on the findings from some recent studies, cfDNA is likely to have a role in detecting minimal residual disease and emerging therapy resistance, in early-stage BC [8]. Moreover, while its use in primary screening and/or early-stage disease is still being explored, cfDNA is currently used mostly to discover and track genetic variations in metastatic disease. While studies have persistently observed elevated levels of cfDNA in BC patients when compared to controls of subjects with benign disease [9,10], the studies on its correlation with cancer subtype are limited.

Of late, similar to global trends, the incidence of BC in India has also increased significantly [11]. Based on Globocan data 2020, BC accounted for 13.5% of all cancer cases in India [1]. Given the elevated incidence of BC on one hand, and the elevation of cfDNA levels in BC patients on the other hand, in the current study, we aimed to evaluate the correlation of cfDNA levels with the molecular subtypes of BC.

## Materials and methods

In the current study, we recruited 39 histo-pathologically confirmed BC patients from the Department of Surgical Oncology, Indira Gandhi Institute of Medical Sciences (IGIMS), Patna, Bihar, India, diagnosed between October 2022 and May 2023. All the patients above the age of 18 years and without any previous cancer were invited to participate in the study. Patients who had received any radiotherapy or chemotherapy were excluded from the study. The study was reviewed and approved by the Institutional Ethics Committee of IGIMS (approval number: 517/IEC/2022/IGIMS). Written informed consent was taken from each patient before their enrolment in the study.

Sample collection and processing

A total of 6 ml of venous blood was taken from each patient. Of the sample, 1 ml was taken in an ethylenediamine tetraacetic acid (EDTA) containing collection vial and subjected to a complete blood count (CBC) using a Swelab Alfa Plus 3-part haematology analyzer (Boule Diagnostics, Spånga, Sweden); 4 ml was transferred to clot activated collection vial (F. Hoffmann-La Roche AG, Basel, Switzerland) and allowed to clot followed by the separation of plasma by centrifuging at 8000 revolutions per minute (RPM) for five minutes in a cold centrifuge. The plasma samples were stored at -80 °C for further analysis.

cfDNA isolation and quantification

The cfDNA was isolated from the plasma samples using the MagMAX™ Cell-Free DNA Isolation Kit (Thermo Fisher Scientific Inc., Waltham, Massachusetts, United States). The manufacturer's instructions were carefully followed for the cfDNA isolation. Briefly, the plasma samples were lysed and a binding solution/beads mix was prepared. Then, an appropriate volume of plasma samples was added, and the beads and the plasma samples were thoroughly mixed by swirling or by inverting the tube 10 times. This was followed by vigorous vortexing for 10 minutes.

The tubes were placed on the appropriate DynaMag™ Magnet (Thermo Fisher Scientific Inc.) for five minutes. The supernatant was discarded and the beads were resuspended in 1 mL of MagMAX Cell-Free DNA Wash Solution (Thermo Fisher Scientific Inc.). The bead slurry was transferred to a new non-stick 1.5-mL microcentrifuge tube and the lysis/binding tube was saved. The microcentrifuge tube containing the bead slurry was placed on the DynaMag-2 Magnet (Thermo Fisher Scientific Inc.) for 20 seconds. The supernatant of the bead slurry was collected and used to rinse the saved lysis/binding microcentrifuge tube. Any residual beads were transferred to the tube containing the bead slurry and the lysis/binding tube was discarded. The tube was left on the DynaMag-2 Magnet for an additional two minutes and the beads were pelleted against the magnets. Then the supernatant was removed with a 1 mL pipette. Keeping the tube on the DynaMag-2 Magnet, the magnet stand was tapped on the benchtop five times, and any residual liquid was removed with a 200-μL pipette. The tube from the DynaMag-2 Magnet was removed and 1 mL of MagMAX Cell-Free DNA Wash Solution was added and vortexed for 30 seconds. This process was repeated three times. CfDNA was then eluted in MagMAX Cell-Free DNA Elution Solution (Thermo Fisher Scientific Inc.). The isolated cfDNA was quantified with a Qubit™ dsDNA high-sensitivity assay kit and the Qubit 4.0 fluorometer (Thermo Fisher Scientific Inc.) following the manufacturer's guidelines.

Statistical analysis

Al the data was recorded in Microsoft Excel (Microsoft Corporation, Redmond, Washington, United States). The continuous variables were presented as mean±SD. Median and centiles were also estimated for the data. One-way ANOVA was used to test the difference between more than two groups. Pearson correlation was also estimated between cfDNA levels and various CBC indices. The coefficient of correlation and respective p-vales were presented in the heatmap. All the data were analysed by GraphPad Prism Version 8 (Insightful Science, LLC, San Diego, California, United States). A two-tailed p-value of the magnitude of <0.05 was considered statistically significant.

## Results

In the current study, we recruited 39 patients who were diagnosed with histopathologically confirmed BC. The mean age of the patients was 48.6±8.20 years. When the subjects were stratified based on immunohistochemistry reports, about 39% (n=16) of subjects were positive for estrogen receptor (ER+), 33.3% of subjects (n=14) were positive for human epidermal growth factor receptor 2 (HER2+), while about 23% (n=9) of subjects belonged to triple-negative BC (TNBC) subtype. The mean levels of cfDNA were 2.81±2.39 ng/µL. The mean levels of various cell types along with their respective 2.5, 50, and 97.5 centiles are also presented in Table 1.

**Table 1:** Mean, median, and centiles of various constituents of the complete blood count, age, and cfDNA levels obtained from patients. WBC: white blood cells, IG: Immunoglobulin, RBC: red blood cells; RDW-CV: red cell distribution width-cell volume; PDW: platelet distribution width; PCV: packed cell volume; MCV: mean corpuscular volume; MCH: mean corpuscular haemoglobin; MCHC: mean corpuscular haemoglobin concentration; PCT: plateletcrit; MPV: mean platelet volume; CHCM: cellular haemoglobin concentration mean; NRBC: nucleated red blood cells; LUC: Large unstained cells; HDW: haemoglobin distribution width; cfDNA: cell-free DNA

Variable	Mean ±SD (N=39)	Percentile		
		2.5^th^	Median	97.5^th^
Age (Years)	48.6±8.2	30	50	60
cfDNA (ng/μL)	2.81±2.39	0.87	1.99	11.2
Haemoglobin	11.4±1.73	7.5	11.5	15
WBC	7.91±2.39	4	7.65	14.6
Neutrophil	61.8±10.7	34.3	63	82.6
Lymphocyte	29±9.27	13.5	28	52
Monocyte	4.56±2.46	0	4.4	10.9
Eosinophil	3.94±4.82	0	2.9	25.6
Basophil	0.31±0.299	0	0.2	0.9
IGg	0.32±0.13	0.1	0.4	0.4
RBC count	4.3±1.19	2	4.25	9.48
Platelet count	213±106	2.37	182	495
RDW-SD	47.5±7.72	35.2	46.1	64.7
RDW-CV	15.5±2.4	12.7	14.8	22.9
PDW	28.1±15.4	10	20.3	61.7
Haematocrit (PCV)	35.8±4.47	24.8	36.2	42.3
MCV	85.5±7.47	61.8	85.3	100
MCH	27.3±2.8	19.3	27.5	31.8
MCHC	31.9±1.52	27.4	32.1	34.3
PCT	0.29±0.11	0.15	0.261	0.6
MPV	11.5±2.02	8.5	11.7	14.9
CHCM	32.6±0.62	31.9	32.4	33.6
NRBC	0.01±0.03	0	0	0.1
LUC	1.7±0.96	0	1.75	3.3
HDW	2.9±0.37	2.54	2.87	3.65

**Figure 1:**
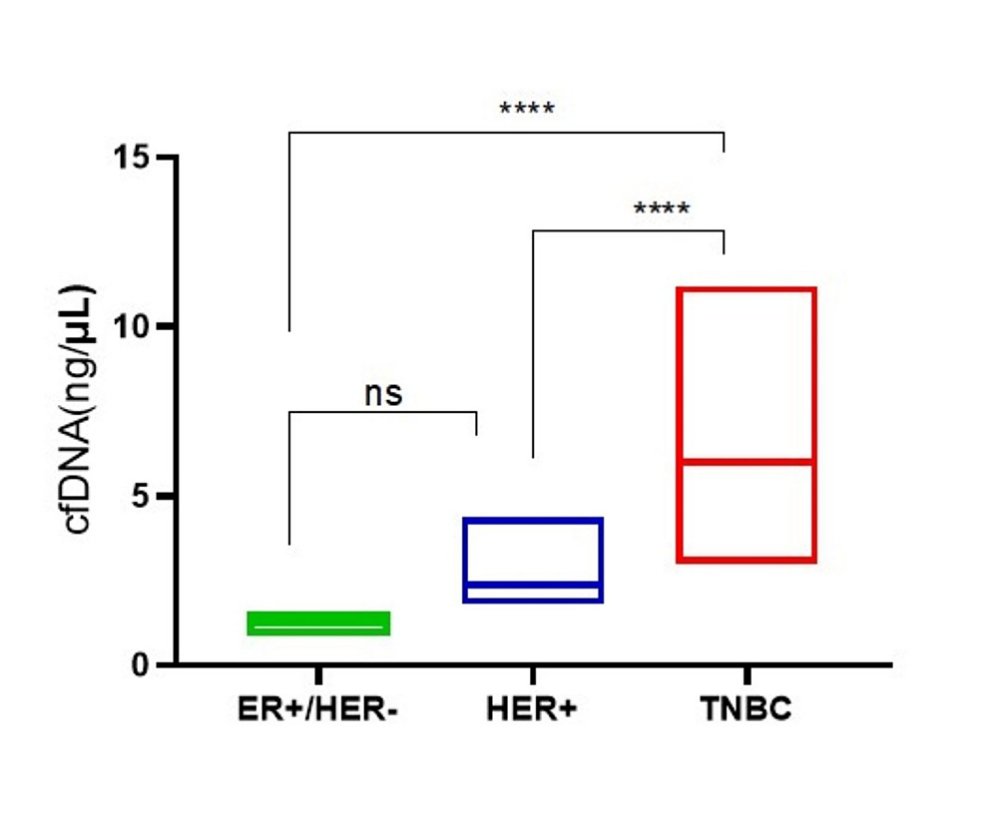
Correlation between the levels of cfDNA and breast cancer subtypes. The levels of cfDNA of the subjects stratified by cancer subtype were plotted in a box-plot. One-way ANOVA was used to test the differences between the groups. ER+: estrogen receptor-positive; HER: human epidermal growth factor; TNBC: triple-negative breast cancer **** p<0.001, ns: non-significant

The levels of cfDNA were significantly higher in patients with the TNBC subtype when compared to ER+ and/or HER2+ patients (p<0.001) (Figure 1).

**Figure 2:**
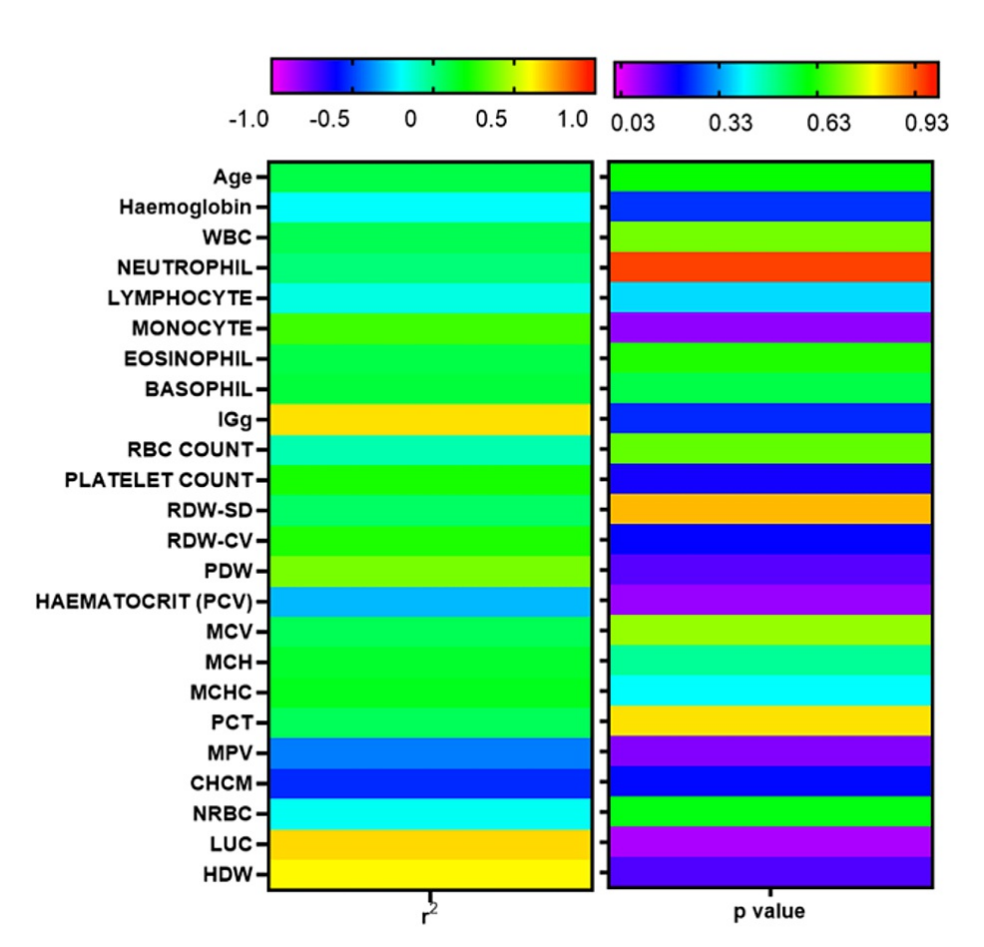
Correlation between the levels of cfDNA and various constituents of blood. The Pearson coefficient of correlation (r2) and respective p-values are plotted in the heat map. WBC: white blood cells; IGg: immunoglobulin gamma; RBC: red blood cells; RDW-CV: red cell distribution width-cell volume; PDW: platelet distribution width; PCV: packed cell volume; MCV: mean corpuscular volume; MCH: mean corpuscular haemoglobin; MCHC: mean corpuscular haemoglobin concentration; PCT: plateletcrit; MPV: mean platelet volume; CHCM: cellular haemoglobin concentration mean; NRBC: nucleated red blood cells; LUC: Large unstained cells; HDW: haemoglobin distribution width; cfDNA: cell-free DNA

No significant correlation was observed between the levels of cfDNA and various indices of the complete blood count (p>0.05) (Figure 2).

## Discussion

In the current study, we observed detectable levels of cfDNA in all the BC patients. As compared to the ER+ patients, cfDNA levels were significantly higher in the patients with either HER2+ or TNBC subtype. However, we did not observe any correlation of cfDNA levels with the constituents of the CBC. Although cfDNA assays for early-stage breast tumours are still in their infancy, recently, there has been a noticeable increase in the number of technologies devoted to early-stage cancer and lower detection limits [12].

The mean age at the time of the disease presentation was ~49 years in the present study. Studies have shown that more than 40% of affected BC patients are usually >65 of age and this age group accounts for around 60% of the total BC-related deaths [13,14]. Moreover, it has been estimated that the risk of developing BC before 49 years is 1/53 while it is 1/43 for the 50-59 years age group. This risk rises to 1/23 for the 60-69 years of age group and is significantly higher in women aged >70 years [14]. It is noteworthy that limited research has focused on the effects of age on the pathogenicity of BC and its subtypes. Moreover, being difficult to mimic in animal models, reproductive status, menarche, and menopause also add to the complexity of understanding the role of age in BC [15]. Therefore, these key unanswered questions warrant further research [16].

We observed the levels of cfDNA were higher in TNBC patients compared to ER+ and HER2+ patients. We speculate that the lower levels of cfDNA in HR+ breast cancer compared with HER2+ and TNBC can be partly due to the lower cellular proliferation rates in this subtype. Our results are in agreement with studies by Magbanua et. al. (for BC) and Abbosh et. al. (for lung cancer) [17,18]. Similar to our findings, Magbanua et al. in their 2023 study also observed that cfDNA levels remained significantly higher in TNBC patients [19]. Moreover, the aggressiveness of the TNBC subtype might also be contributing to the elevated levels of cfDNA levels in such patients [20]. Understanding the molecular and genetic factors that indicate the presence of cfDNA in the blood may provide insight into the biology of cfDNA presence and its clearance during therapy [17]. These findings are suggestive that early response prediction by very sensitive cfDNA analysis in high-risk early-onset BC patients may enable a prompt and judicious shift in treatment to enhance the treatment modalities for better outcomes for the patients.

The diagnostic value of cfDNA from the blood of cancer patients was first studied by Leon et al. by observing an association of cfDNA levels with tumour burden, response to therapy, and prognosis [21]. Huang et al. corroborated these results and found that, unlike healthy controls, cfDNA levels were five-fold higher in BC patients, while the levels cfDNA in BC patients were approximately three-fold higher than in patients with benign breast tumours [22]. The results from these pioneering studies pointed out the diagnostic value of cfDNA for the early detection of BC. A recent comparative study also observed that cfDNA levels were higher in BC patients before surgery when compared with their levels post-surgery [23].

Despite the fact that several studies have reported that the level of cfDNA is elevated in the blood of cancer patients, defining a diagnostic threshold has remained elusive so far [12]. Besides, certain biological processes and diseases other than cancer can also significantly modulate the levels of cfDNA [24,25]. Moreover, studies on the differences between levels of cfDNA in patients with benign vs malignant disease have shown mixed results with a few authors questioning the reliability of cfDNA as a diagnostic tool [26,27]. Nonetheless, many studies have shown elevated levels of cfDNA in BC patients when compared to subjects with benign disease [9,10]. While the study by Elhelaly et al. [9] enrolled benign subjects as controls, the authors did not stratify patients based on molecular subtypes, unlike our study. In the current study, we did not find any correlation between the levels of cfDNA with different cell types constituting blood and other indices of CBC.

Limitations

While ours was the first such attempt, the results are based on a moderate sample size. Moreover, enrolling a control arm with apparently healthy women would have added more confidence to the data presented in the presented study. Therefore, more replicative studies with a large sample size are warranted to substantiate our results.

## Conclusions

cfDNA was found in a detectable range in all the BC patients. Varying levels of cfDNA in different molecular and histopathological subtypes of BC observed in the study indicate a potential association of cfDNA levels with the subtypes of BC. To support our findings, more replicative studies are warranted.

## References

[1] Sung H, Ferlay J, Siegel RL, Laversanne M, Soerjomataram I, Jemal A, Bray F (2021). Global cancer statistics 2020: GLOBOCAN estimates of incidence and mortality worldwide for 36 cancers in 185 countries. CA Cancer J Clin.

[2] Unger-Saldaña K (2014). Challenges to the early diagnosis and treatment of breast cancer in developing countries. World J Clin Oncol.

[3] Trayes KP, Cokenakes SE (2021). Breast cancer treatment. Am Fam Physician.

[4] Barrios CH (2022). Global challenges in breast cancer detection and treatment. Breast.

[5] Halim AA, Andrew AM, Yasin MN (2021). Existing and emerging breast cancer detection technologies and its challenges: a review. Appl Sci.

[6] Zardavas D, Phillips WA, Loi S (2014). PIK3CA mutations in breast cancer: reconciling findings from preclinical and clinical data. Breast Cancer Res.

[7] Rossi G, Ignatiadis M (2019). Promises and pitfalls of using liquid biopsy for precision medicine. Cancer Res.

[8] Garcia-Murillas I, Chopra N, Comino-Méndez I (2019). Assessment of molecular relapse detection in early-stage breast cancer. JAMA Oncol.

[9] Elhelaly R, Effat N, Hegazy MA, Abdelwahab K, Hamdy O, Abo Hashem EM, Elzehery RR (2022). Circulating cell free DNA and DNA integrity index as discriminating tools between breast cancer and benign breast disease. Asian Pac J Cancer Prev.

[10] El Edel RH, El Gamaal AS, El-Said HH, Noreldin RI, Omar MM (2018). Cell-free DNA as a biomarker of breast cancer. Menoufia Med J.

[11] Mehrotra R, Yadav K (2022). Breast cancer in India: present scenario and the challenges ahead. World J Clin Oncol.

[12] Croessmann S, Park BH (2021). Circulating tumor DNA in early-stage breast cancer: new directions and potential clinical applications. Clin Adv Hematol Oncol.

[13] DeSantis C, Siegel R, Bandi P, Jemal A (2011). Breast cancer statistics, 2011. CA Cancer J Clin.

[14] Siegel R, Ma J, Zou Z, Jemal A (2014). Cancer statistics, 2014. CA Cancer J Clin.

[15] Warner ET, Colditz GA, Palmer JR, Partridge AH, Rosner BA, Tamimi RM (2013). Reproductive factors and risk of premenopausal breast cancer by age at diagnosis: are there differences before and after age 40?. Breast Cancer Res Treat.

[16] McGuire A, Brown JA, Malone C, McLaughlin R, Kerin MJ (2015). Effects of age on the detection and management of breast cancer. Cancers (Basel).

[17] Magbanua MJ, Swigart LB, Wu HT (2021). Circulating tumor DNA in neoadjuvant-treated breast cancer reflects response and survival. Ann Oncol.

[18] Abbosh C, Birkbak NJ, Wilson GA (2017). Phylogenetic ctDNA analysis depicts early-stage lung cancer evolution. Nature.

[19] Magbanua MJ, Brown Swigart L, Ahmed Z (2023). Clinical significance and biology of circulating tumor DNA in high-risk early-stage HER2-negative breast cancer receiving neoadjuvant chemotherapy. Cancer Cell.

[20] Orrantia-Borunda E, Anchondo-Nuñez P, Acuña-Aguilar LE, Gómez-Valles FO, Ramírez-Valdespino CA (2022). Subtypes of breast cancer. Breast Cancer.

[21] Leon SA, Shapiro B, Sklaroff DM, Yaros MJ (1977). Free DNA in the serum of cancer patients and the effect of therapy. Cancer Res.

[22] Huang ZH, Li LH, Hua D (2006). Quantitative analysis of plasma circulating DNA at diagnosis and during follow-up of breast cancer patients. Cancer Lett.

[23] Agassi R, Czeiger D, Shaked G (2015). Measurement of circulating cell-free DNA levels by a simple fluorescent test in patients with breast cancer. Am J Clin Pathol.

[24] Gahan PB, Swaminathan R (2008). Circulating nucleic acids in plasma and serum. Recent developments. Ann N Y Acad Sci.

[25] Fournié GJ, Courtin JP, Laval F (1995). Plasma DNA as a marker of cancerous cell death. Investigations in patients suffering from lung cancer and in nude mice bearing human tumours. Cancer Lett.

[26] Rohanizadegan M (2018). Analysis of circulating tumor DNA in breast cancer as a diagnostic and prognostic biomarker. Cancer Genet.

[27] Zanetti-Dällenbach RA, Schmid S, Wight E, Holzgreve W, Ladewing A, Hahn S, Zhong XY (2007). Levels of circulating cell-free serum DNA in benign and malignant breast lesions. Int J Biol Markers.

